# Chemerin-Derived Peptide Val^66^-Pro^85^ Is Effective in Limiting Methicillin-Resistant *S. aureus* Skin Infection

**DOI:** 10.3389/fmicb.2021.742610

**Published:** 2021-11-03

**Authors:** Aneta Zegar, Urszula Godlewska, Dorota Kozłowska-Chmielewska, Pawel Majewski, Brian A. Zabel, Joanna Cichy

**Affiliations:** ^1^Department of Immunology, Faculty of Biochemistry, Biophysics and Biotechnology, Jagiellonian University, Kraków, Poland; ^2^Palo Alto Veterans Institute for Research, VA Palo Alto Health Care System, Palo Alto, CA, United States

**Keywords:** antimicrobial peptides, chemerin, skin, MRSA—methicillin-resistant *Staphylococcus aureus*, atopic dermatitis

## Abstract

Chemerin-derived peptide Val^66^-Pro^85^ (p4) restricts the growth of a variety of skin-associated bacteria, including methicillin-resistant *Staphylococcus aureus* (MRSA). To better understand the antimicrobial potential of chemerin peptide, we compared p4 activity against MRSA *in vitro* to cathelicidin LL-37, one of the key endogenous peptides implicated in controlling the growth of *S. aureus*. The efficacy of p4 was also validated in relevant experimental models of skin pathology, such as topical skin infection with community-acquired MRSA, and in the context of skin inflammatory diseases commonly associated with colonization with *S. aureus*, such as atopic dermatitis (AD). We showed that p4 collaborates additively with LL-37 in inhibiting the growth of *S. aureus*, including MRSA, and that p4 was effective *in vivo* in reducing MRSA burden. p4 was also effective in reducing levels of skin-infiltrating leukocytes in *S. aureus*-infected AD-like skin. Taken together, our data suggest that p4 is effective in limiting *S. aureus* and, in particular, MRSA skin infection.

## Introduction

Chemerin is a potent protein attractant for several leukocyte subsets, including dendritic cells, macrophages, and an adipokine implicated in metabolic regulation (Wittamer et al., 2003; Zabel et al., 2005b, 2014; Goralski et al., 2007).

Chemerin bactericidal potential and expression by the skin also suggest that chemerin plays a direct role in shaping resident cutaneous bacterial communities and can limit skin infection. Recombinant chemerin exhibits antimicrobial activity *in vitro* against various bacterial strains of the commensal skin microbiome (Kulig et al., 2011; Banas et al., 2013; Godlewska et al., 2019, 2020). Moreover, human epidermal chemerin is largely responsible for the natural antimicrobial activity present in keratinocyte secretions (Banas et al., 2013). In addition, genetic ablation of the chemerin gene *RARRES2* can result in higher counts of viable epidermal bacteria in an experimental model of skin infection (Banas et al., 2015). Finally, epidermal downregulation of chemerin in psoriasis correlates with some changes in the skin microbiome (Godlewska et al., 2020). Since chemerin is downregulated in psoriasis and is bactericidal against certain bacteria and not others, it is possible that chemerin downregulation contributes to alterations in the skin microbiome during psoriasis (Godlewska et al., 2020). Chemerin may therefore play a beneficial role in normal healthy epidermis as a natural antibiotic-like molecule.

Therapeutic application of endogenous antimicrobial factors such as LL-37 can effectively restrict the growth of pathogenic cutaneous microorganisms (Duplantier and van Hoek, 2013). Chemerin-derived Val^66^-Pro^85^ peptide (p4, Table 1) embodies the majority of chemerin’s antimicrobial activity (Banas et al., 2013). The 20-amino acid-long p4 chemerin peptide displays similar antimicrobial activities as active chemerin, although with increased efficacy and potency (Banas et al., 2013; Godlewska et al., 2017, 2019, 2020).

**TABLE 1:** Sequences of peptides used in the experiments.

**Peptide name**	**Sequence**
p4	VRLEFKLQQTSCRKRDWKKP
Scramble p4 (scp4)	DPWLKVRKFQTLKQREKRCS

*Staphylococcus aureus* is among the most common causes of bacterial infections in humans, including skin infection. *S. aureus* strains such as USA300 pose an antibiotic resistance threat, as they have been documented to acquire gene cassettes encoding resistance to methicillin and other frontline antibiotics (Carrel et al., 2015). Synthetic p4 is effective against various *S. aureus* strains, including MRSA strains BAA-1707 and E240 *in vitro* (Godlewska et al., 2019). Moreover, topical treatment with p4 significantly suppressed the growth of the *S. aureus* laboratory strain 8325-4 in an experimental model of skin infection (Godlewska et al., 2019).

Here, we further investigated the antimicrobial potential of p4 as it related to two clinically relevant *S. aureus*-mediated skin pathologies: health care-associated MRSA infection and chronic inflammatory skin disease atopic dermatitis (AD), where *S. aureus* contributed to disease pathology (Williams et al., 2017).

## Materials and Methods

### Bacterial Strains

The bacterial strains used in the study were the conventional laboratory strain *S. aureus* 8325-4 and MRSA strain *S. aureus* USA300 (ATCC BAA*-*1717). The MRSA strain was kindly donated by Dr. A. Sabat (University of Groningen, Groningen, Netherlands).

### Peptides

Peptide p4 and a control peptide scp4 (Table 1) were chemically synthesized by ChinaPeptides (Shanghai, China) at ≥ 95% purity. LL-37 was obtained from Innovagen (Sweden).

### Antimicrobial Microdilution Assay and Checkerboard Assay

For all *in vitro* experiments, bacteria were cultured in tryptic soy broth (TSB) (Sigma-Aldrich) under aerobic condition. To determine the antimicrobial activity of LL-37 and peptide p4, bacteria in the mid-logarithmic phase were diluted to 4 × 10^5^ colony-forming units (CFUs)/ml with PBS containing a series of twofold dilution of peptide or PBS (control) and incubated for 2 h. To test the antimicrobial activity of a combination of antimicrobial peptides (AMPs), bacteria were incubated with LL-37 (0.6 μM) and/or p4 (1.6 μM) for 2 h. The number of viable bacteria were enumerated by CFU counting. *N* = 3 independent experiments. To determine whether LL-37 and p4 exhibit antimicrobial synergy against USA300, we performed a checkerboard assay and calculated the fractional inhibitory concentration (FIC) index value as previously described (Joung et al., 2015). The synergy tests were performed using peptides in the predetermined range of twofold dilution: LL-37 (0.08–20 μM) and p4 (0.8–50 μM) incubated with bacteria for 2 h. The minimum inhibitory concentration (MIC) was defined as the lowest concentration of each peptide alone or in their combination that resulted in no visible growth. To determine the impact on potency of the combination of p4 and LL-37 in comparison to their individual activities, the FIC index value was calculated using the following formula: MIC of LL-37 in combination with p4/MIC of LL-37 alone + MIC of p4 in combination with LL-37/MIC of p4 alone. The FIC index value was interpreted as follows: < 0.5, synergy; 0.5–4, additive or indifference; and > 4 antagonism. *N* = 2 independent experiments, each performed with three biological replicates.

### Topical Skin Infection

Female or male 8–12-week-old C57BL/6 mice and chemerin-deficient mice on C57BL/6 background (ChemKO) (Banas et al., 2015) were housed under pathogen-free conditions in the animal facility at the Faculty of Biochemistry, Biophysics, and Biotechnology of Jagiellonian University. Chemerin is encoded by TIG2 (tazarotene-induced gene 2), also known as RARRES2 (retinoic acid receptor responder gene 2), and ChemKO mice are deficient in the Tig2 gene (Banas et al., 2015). All animal studies were approved and in compliance with the guidelines of the Second Local Ethical Committee on Animal Testing at the Institute of Pharmacology Polish Academy of Sciences in Krakow (#298/2017 and 103/2019). A small dorsal area of the skin was shaved, sterilized with ethanol and punctured six times at two places using a syringe needle (BD Micro-Fine Plus, 0.3 × 8 mm). Two 8-mm-inner diameter rubber rings were subsequently attached using an ethyl cyanoacrylate-based adhesive, and peptides or vehicle were topically administered in mouse skin. The peptides were allowed to dry on the skin, and the rings were covered with OpSite (Smith and Nephew). *S. aureus* of 1 × 10^7^ CFU in a volume of 50 μl was thereafter injected through the OpSite into the cavity formed by the rubber rings. The ring injected with sterile PBS was used as control. After 24 h, bacterial loads were analyzed by enumeration of CFU. The skin within the side of the rings was retrieved, frozen, and fixed in methanol for 1 min followed by Gram staining (Fluka). For *ex vivo* experiments, skin biopsies were treated with vehicle or peptides followed by infection with *S. aureus*. Each independent experiment involved one to three mice per experimental group, and one to two treatment sites (two independent cavities) per mouse.

### Experimental Model of Atopic Dermatitis

An AD-like experimental model was induced as previously described (Jin et al., 2009) with minor modifications. Briefly, 6-week-old C57BL/6 mice were shaved on dorsal skin and 24 h later subjected to type stripping 6x with a Polopor adhesive tape (Viscoplast, 3M). Ovalbumin (OVA) of 100 μg or vehicle control (PBS) of 100 μl was applied to a sterile patch which was in direct contact with the skin surface and which was secured to the skin with a transparent, adhesive dressing. The patch was placed on the skin twice for a 1-week treatment with a 2-week interval before a second OVA or PBS application. One week after the last application of OVA or PBS, mice were subjected to topical skin infection as described above.

### ELISA

Total IgE and OVA-specific IgE serum levels were quantified using a sandwich and direct ELISA, respectively. For detection of total IgE, strips (MaxiSorp Nunc-Immuno Module, Thermo Scientific) were coated with 2 μg/ml of rat anti-mouse IgE mAb (BD Pharmingen) in Tris-buffered saline (50 mM Tris-HCl, pH 9.5, 150 mM NaCl). The strips were then washed with 0.1% Tween 20, and non-specific protein-binding sites were blocked with 3% bovine serum albumin (BSA). Serum samples were added and incubated at room temperature (RT) for 1 h. Purified mouse IgE (BD Pharmingen) was used as a standard. Bound IgE was detected using FITC-anti-IgE (BioLegend) incubated at RT for 1 h followed by incubation with horseradish peroxidase (HRP)-conjugated anti-FITC mAb (Thermo Scientific) and developed with a TMB substrate (BD Biosciences).

To detect OVA-specific IgE, strips were coated with OVA (10 μg/ml, Sigma). A strip was washed and blocked using 3% BSA. Afterward, serum was applied and incubated for 2 h at room temperature. A plate was washed, and OVA-specific IgE antibodies were detected using FITC-conjugated mAb as described above. For positive control, we included the well-established model of OVA-induced IgE response (Kips et al., 2003).

The positive control represents sera pooled from five C57BL/6 mice that were immunized intraperitoneally (i.p.) with 10 μg OVA in the presence of 100 μg alum adjuvant (Thermo Fisher Scientific) for 10 days.

### Immunohistochemistry

Frozen 8-μm sections were prepared from skin biopsies. Sections were fixed in acetone, blocked with 3% BSA (Sigma), and then stained with APC-labeled rat anti-mouse CD45 (BioLegend) mAb or isotype control APC-labeled rat IgG2b (BioLegend). The sections were counterstained with Hoechst 33258 (Invitrogen). Images were captured with a fully motorized fluorescence microscope (NIKON, Eclipse) and analyzed by NIS elements software (Nikon). For each mouse (three mice per group from three independent experiments), 15 different high-power fields spanning the epidermis and dermis were analyzed.

### RT-qPCR

Total RNA was extracted with the Total RNA Zol-Out Kit (A&A Biotechnology) and converted to cDNA using NxGen^TM^ M-MuLV reverse transcriptase (Lucigen) with a mix of random hexamers (Invitrogen) and oligo (dT) (GenoMed). Real-time PCR was performed on the CFX96 thermocycler (Bio-Rad Laboratories) using SYBR Green I containing a Universal PCR Master Mix (A&A Biotechnology) and primers specific for mouse camp (5′-CTTCAAGGAACAGGGGGTGG-3′, 5′-ACCTTTGCGGAGAAGTCCAG-3′) and two housekeeper genes B2M (5′-GGACTGGTCTTTCTATATCCTGGC-3′, 5′-GA TCACATGTCTCGATCCCAGTAG-3′) and GAPDH (5′-TGT GTCCGTCGTGGATCTGA-3′, 5′-TTGCTGTTGAAGTCGCA GGAG-3′). Gene expression normalized to geometric mean of the housekeeper genes was calculated using the 2^–ΔCt^ method (Vandesompele et al., 2002; Nessi et al., 2010).

### Statistical Analysis

Statistical analyses were performed using Statistica (StatSoft, Dell software) and GraphPad Prism (GraphPad software). Data were presented as mean ± standard deviation (SD). For multiple comparisons, either one-way ANOVA followed by a Tukey *post hoc* test or Kruskal–Wallis test with Dunn’s multiple comparison *post hoc* test was used. The unpaired two-tailed *t*-test or Mann–Whitney test was performed for comparison between two groups. In order to compare the study group with the control group, the one-sample *t*-test or its nonparametric alternative, the one-sample Wilcoxon test, was used. Differences were considered statistically significant when *p* < 0.05.

## Results

**FIGURE 1:**
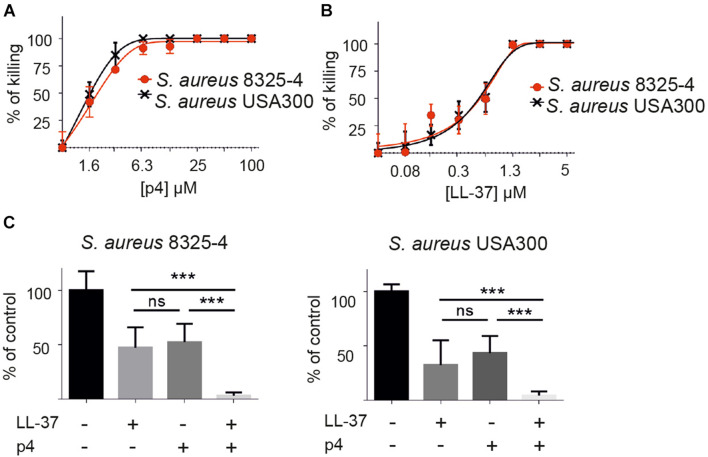
p4 is bactericidal *in vitro* against different strains of *S. aureus* and collaborates additively with peptide LL-37 in restricting *S. aureus* growth. **(A)** *S. aureus* strains 8325-4 and USA300 were incubated with the indicated concentration of p4 for 2 h. Cell viability in CFU/ml, shown as the percentage of killing for the indicated strain, was analyzed by MDA assay. **(B)** The indicated *S. aureus* strains were incubated with the indicated concentration of LL-37 for 2 h. Cell viability in CFU/ml, shown as the percentage of killing for the indicated strain, was analyzed by MDA assay. **(C)** The indicated *S. aureus* strains were incubated with 1.6 μM p4 and/or 0.6 μM LL-37 for 2 h. Cell viability in CFU/ml, shown as the percentage of control cells (bacteria incubated with PBS), was analyzed by MDA assay. Results are expressed as the mean ± SD of three independent experiments. ***p < 0.0001, ns, non-significant by one-way ANOVA with *post hoc* Tukey’s multiple comparison test.

We first assessed p4 bactericidal activity *in vitro* against two strains of *S. aureus*: laboratory strain 8325-4 (Godlewska et al., 2019, 2020) and *S. aureus* USA300, the causative strain of the most common community-associated MRSA infections (Carrel et al., 2015). p4 restricted the growth of both strains in a similar fashion (Figure 1A). Next, we compared the anti-staphylococcal activities of p4 and peptide LL-37, a 37-amino acid C-terminal derivative of human cathelicidin hCAP18. Bioactive cathelicidin derivatives are among the most important endogenous skin antimicrobial peptides and are highly protective against invasive *S. aureus* skin infection (Zhang et al., 2015). While both p4 and LL-37 could completely abrogate the growth of both *S aureus* strains, p4 was less potent than LL-37, MIC = 25 and 2.6 μM, respectively (Figures 1A,B). Of note, when both peptides were tested at sublethal concentrations (∼50% growth inhibition), p4 (1.6 μM) displayed additive effects with LL-37 (0.6 μM) in inhibiting the growth of *S. aureus* 8325-4 and *S. aureus* USA300 (Figure 1C). The additive but not synergistic interaction between p4 and LL-37 against *S. aureus* USA300 was confirmed by checkerboard assay, with the FIC index value for tested peptides being equal to 1.004 (where a FIC index < 0.5 indicates synergy and 0.5–4 indicates potential additive effects) (Joung et al., 2015). Together, these data suggest that p4 may be useful therapeutically against *S. aureus* strains resistant to common antibiotics and acts additively with endogenous LL-37 to suppress bacterial growth in infected skin.

**FIGURE 2:**
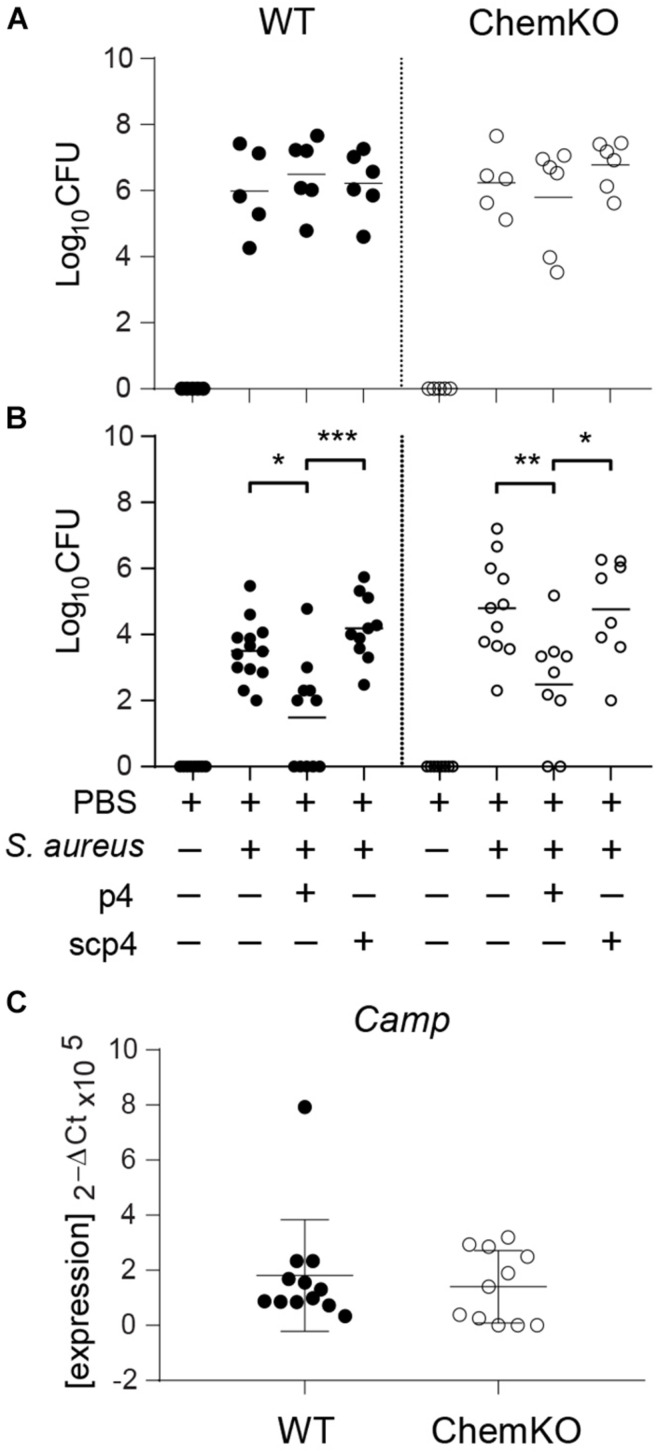
p4 limits experimental *S. aureus* infection *in vivo*. WT and ChemKO mice were treated with vehicle (PBS) or were topically infected with 1 × 10^7^ CFU of *S. aureus* 8325-4 in the presence of 100 μM p4, control peptide scp4, or PBS. **(A)** Data points indicate the number of bacteria recovered from the skin surface 3 h after application of bacteria. **(B)** Data points indicate the number of live bacteria recovered from the skin surface 24 h after application of bacteria. **(C)** Data points indicate *Camp* expression levels in the skin treated with PBS for 24 h. Each data point represents one cavity, and a horizontal line indicates the mean value in each group; *n* = 5–10 independent experiments (1–2 mice per group). ***p < 0.001; **p < 0.01; *p < 0.05 by the Kruskal–Wallis test with *post hoc* Dunn’s multiple comparisons test and one-way ANOVA with Tukey’s *post hoc* test.

To assess whether p4 can reduce skin colonization by *S. aureus in vivo*, we applied p4 or control scramble peptide 4 (scp4) (Table 1) to denuded mouse skin and then challenged the site with *S. aureus* 8325-4 or PBS control (to control for any residual commensal bacteria). Bacterial loads recovered from the skin surface 3 and 24 h later were determined by CFU quantification (Figure 2). We also treated chemerin-deficient (ChemKO) mice with p4 to ask if the peptide had anti-staphylococcal activity in the presence or absence of endogenous chemerin in the skin. Whereas 3-h treatment of *S. aureus* with p4 on the skin did not lead to any significant reduction in cutaneous *S. aureus* burden when compared to scp4-treated wild-type (WT) and ChemKO mice (Figure 2A), both WT and ChemKO mice had significantly lower *S. aureus* loads when treated with p4 but not scp4 for 24 h (Figure 2B). These data are consistent with our previously published results on WT mice topically infected with *S. aureus* 8325-4 and treated with the peptides for 24 h (Godlewska et al., 2019). However, p4 appeared to be less effective in controlling *S. aureus* growth in ChemKO compared with WT mice, reducing *S. aureus* overall abundance from 100% to 54.4 ± 34.6% vs. 38.6 ± 40.1 (mean ± SD) in ChemKO and WT mice, respectively (Table 2). These data suggest that deficiency in chemerin might render mice less susceptible to p4 anti-staphylococcal activity. Since LL-37 potentiates p4 effect *in vitro* (Figure 1C), it was possible that mice null of chemerin produce less cathelicidin, and therefore, p4 cannot fully collaborate additively with the mouse analog of LL-37 (protein CRAMP) to efficiently decrease staphylococcal relative abundances. However, qPCR for skin expression of mouse cathelicidin gene *Camp* that encodes CRAMP did not reveal a significant difference between WT and ChemKO mice (Figure 2C).

**TABLE 2:** WT and ChemKO mice (*in vivo*) or skin biopsies taken from these mice (*ex vivo*) were challenged with 1 × 10^7^ CFU of *S. aureus* 8325-4 in the presence of vehicle (PBS) (control), 100 μM p4, or scp4 for 24 h.

		**WT [%]**	**ChemKO [%]**
*In vivo*	*S. aureus* PBS (control)	100	100
	*S. aureus* + p4	38.6 ± 40.1**	54.4 ± 34.6**
	*S. aureus* + scp4	126.6 ± 40.7	122.1 ± 40.9
*Ex vivo*	*S. aureus* PBS (control)	100	100
	*S. aureus* + p4	88.3 ± 4.7**	91.3 ± 4.4*

Data indicate percentage of recovered bacteria relative to control (mean ± SD), n = 8–11 (in vivo) or 5 (ex vivo) independent experiments (1–2 mice per experiment). **p < 0.01; *p < 0.05 by the one-sample Wilcoxon test and one-sample t-test, comparing p4 vs. control.

**FIGURE 3:**
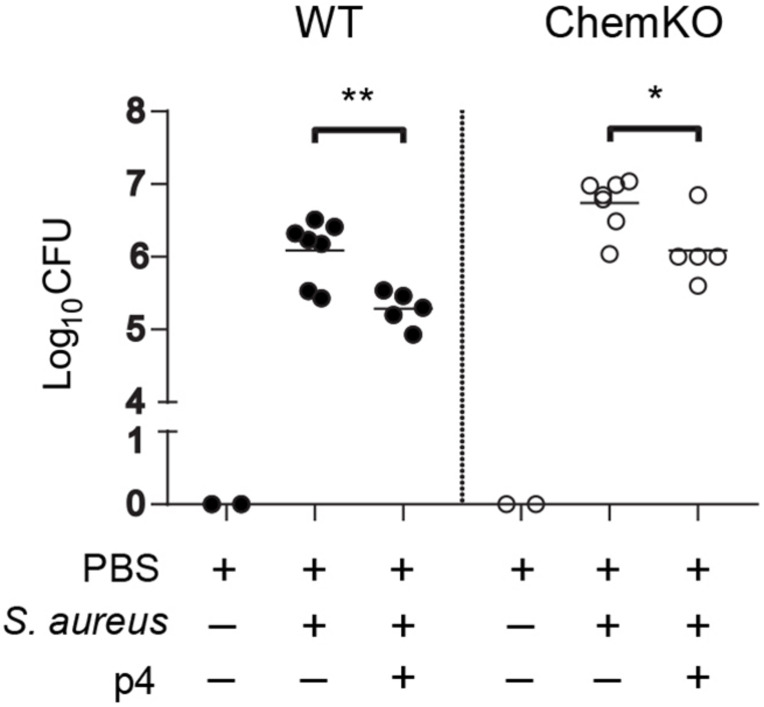
p4 is bactericidal *ex vivo*. Skin biopsies from WT and ChemKO mice were challenged with 1 × 10^7^ CFU of *S. aureus* 8325-4 in the presence of 100 μM p4 or vehicle (PBS). Data points indicate the CFU of bacteria recovered from the skin surface 24 h after application of bacteria, with each data point representing one cavity and a horizontal line indicating the mean value in each group; *n* = 5 (1–2 mice per group). **p < 0.01; *p < 0.05 by unpaired *t*-test.

To assess whether p4 has primary local antimicrobial effects in the skin or if it requires elaboration of secondary circulating factors (e.g., complement or white blood cells) for maximal activity, we treated skin biopsies from WT and ChemKO mice *ex vivo* with p4 or vehicle followed by infection of the biopsies with *S. aureus* 8325-4 for 24 h. In agreement with the *in vivo* data, application of p4 significantly reduced the *S. aureus* burden compared with vehicle in the skin biopsies derived from both WT and ChemKO mice (Figure 3). Likewise, p4 appeared to be slightly less effective against bacteria in biopsies from ChemKO compared to WT mice (Table 2). These data suggest that while p4 is able to restrict the growth of *S. aureus in situ* and that chemerin can facilitate primary antimicrobial p4 effects by endowing the skin with properties that support local direct p4 anti-staphylococcal action, maximal p4 activity requires additional *in vivo* factors.

**FIGURE 4:**
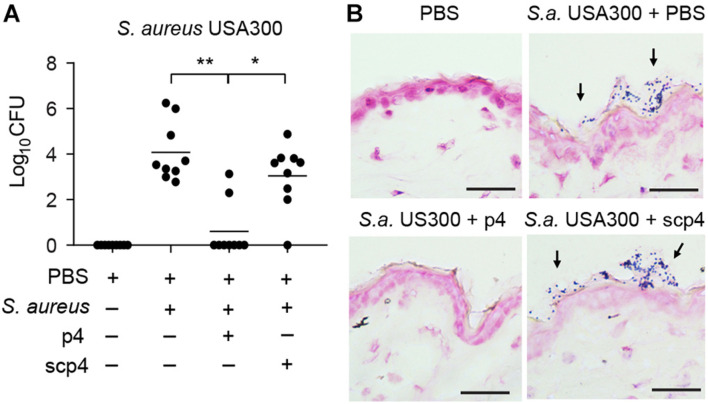
p4 is effective against MRSA in the experimental model of skin infection. **(A)** WT mice were treated with vehicle (PBS) or ectopically infected with 1 × 10^7^ CFU of *S. aureus* USA300 in the presence of 100 μM p4, control peptide scp4, or PBS. Data points indicate the number of live bacteria recovered from the skin surface 24 h after application of MRSA, with each data point representing one cavity and a horizontal line indicating the mean value in each group; *n* = 6. **p < 0.01; *p < 0.05 by the Kruskal–Wallis test with *post hoc* Dunn’s multiple comparisons test. **(B)** Mice were treated as in **(A)** followed by Gram staining. *S. aureus* on the skin surface is indicated by *arrows*. Scale bar = 20 μm.

Given the significant decrease in *S. aureus* 8325-4 abundance *ex vivo* and *in vivo* by p4, we next asked if p4 can control community-acquired MRSA strains on the skin. We topically applied p4 and control scp4 to the skin of WT mice and then challenged with the experimental MRSA strain USA300. Following 24-h incubation, p4 significantly suppressed MRSA growth compared with scp4 or vehicle controls (Figures 4A,B). These data highlight the effectiveness of p4 against clinically relevant antibiotic-resistant community-acquired *S. aureus*.

**FIGURE 5:**
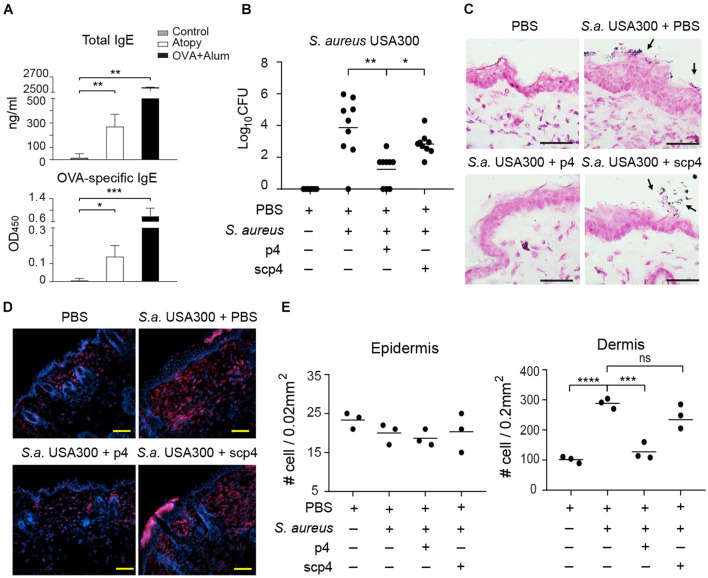
p4 reduces MRSA burden and accompanying skin inflammation in the experimental model of AD. Mice were subjected to tape-striping and OVA challenge to induce an AD-like phenotype (Atopy). **(A)** Allergic changes were monitored by ELISA specific for total or OVA-specific serum IgE levels, upper and lower panels, respectively. The negative control represents sera from sex- and aged-matched unchallenged mice (control). The positive control represents sera of mice immunized i.p. with OVA in the presence of alum adjuvant for 10 days (OVA+Alum). The antibody titers are shown as mean ± SD ng/ml or OD (optical density units) for the indicated sets of sera. *n* = 4–5, ***p < 0.001; **p < 0.01; *p < 0.05 by Kruskal–Wallis test with *post hoc* Dunn’s multiple comparisons test. **(B)** Mice were subjected to tape-striping and OVA challenge, followed by bacterial challenge and/or treatment with the indicated factors. Data points indicate the number of live bacteria recovered from the skin surface 24 h after application of *S. aureus*, *n* = 8. **p < 0.01; *p < 0.05 by the Kruskal–Wallis test with *post hoc* Dunn’s multiple comparison test. **(C)** Representative Gram staining of the indicated skin biopsies from the AD model. *S. aureus* on the skin surface is indicated by *arrows*. **(D)** Representative fluorescence microscope images of the indicated AD skin, stained with Hoechst to detect DNA (blue) and anti-CD45 to identify leukocytes (red). **(E)** Data points indicate # of leukocytes (CD45+) infiltrating the indicated area of AD skin, *n* = 3 mice per group. ****p < 0.0001; ***p < 0.001; ns, not significant; by one-way ANOVA with *post hoc* Dunnett’s multiple comparisons test. Scale bar = 20 μm.

Since excessive skin colonization with *S. aureus* is common in AD and the bacteria potentially play a causative role in the development and/or exacerbation of AD (Nakamura et al., 2013; Nakatsuji et al., 2017; Kim et al., 2019), we next determined whether p4 is effective in the context of AD skin pathology. To develop an AD-like skin phenotype, mice were subjected to mechanical barrier disruption by tape-stripping and repeated application of OVA. To mimic the early stages of AD development in which eczematous flares can be driven by *S. aureus* colonization, mice were subjected to two cycles of epidermal sensitization with OVA or saline control (Jin et al., 2009). The development of AD was indicated by significant increases in total and OVA-specific serum IgE levels compared to PBS-treated controls (Figure 5A), as well as skin alterations such as epidermal thickening and/or immune cell infiltration (Figures 5C–E). *S. aureus* was undetectable in PBS-treated controls (Figures 5B,C), indicating a lack of potentially confounding commensal *S. aureus* on mouse skin. Application of p4 but not scp4 to the skin of mice challenged with *S. aureus* USA300 for 24 h decreased the amount of viable bacteria, although in most experiments it did not eradicate *S. aureus* completely (Figure 5B). p4 treatment significantly reduced skin leukocyte infiltration upon MRSA challenge as detected by CD45 staining and fluorescence microscopy (Figures 5D,E). These data demonstrate that in the setting of experimental AD, p4 can be effective in reducing MRSA burden. The lower relative abundance of USA300 in the p4-treated AD-like skin was accompanied by fewer cutaneous immune cells, suggestive of a reduced pathogenic inflammatory response in p4-treated USA300-infected skin.

## Discussion

Here we report *in vitro*, *ex vivo*, and *in vivo* activities of protective chemerin-derived peptide p4, offering a potential strategy to limit skin infection with *S. aureus* including community-associated MRSA.

p4 is more effective than chemerin as an antimicrobial biologic against *S. aureus* (Banas et al., 2013; Godlewska et al., 2017, 2020), and due to its short length (20 amino acids), p4 can be chemically synthesized for low cost and as a homogenous product. The majority of chemerin functions, such as chemotactic and metabolic activities, require chemerin binding to G-protein-coupled chemokine receptor CMKLR1 (Zabel et al., 2005b, 2014). p4-mediated anti-staphylococcal activity and CMKLR1-mediated chemoattractant activity of chemerin are located in different domains of the protein (Banas et al., 2013; Zabel et al., 2014). Therefore, p4 is not likely to interfere with interactions between chemerin and CMKLR1, allowing chemerin to exert other actions, such as creating an infection-controlling environment through recruiting CMKLR1-expressing leukocytes.

Chemerin appears to directly add to the antimicrobial effect of p4 and/or indirectly facilitate p4 anti-staphylococcal action on the skin, as suggested by more effective control of *S. aureus* growth in WT mice compared to ChemKO mice observed in both *in vivo* and *ex vivo* models of skin infection (Figures 2, 3 and Table 2). One potential explanation of this difference is that ChemKO mice may lack any chemerin-mediated bacterial growth control and gain some of that back by topically applied p4, but there is still a deficit compared to the amount of chemerin- and p4-dependent bacterial growth inhibition in chemerin-replete WT mice. However, the effect of chemerin might also depend on its chemotactic or other activities and at least partly rely on locally produced factors and/or circulating agents that can render the skin more susceptible to the action of p4. These could include other AMPs acting in concert with p4 to contain *S. aureus* growth. Alternatively, p4 may be more effective in the presence of chemerin due to its competition with chemerin substrate as a target for proteolytic enzymes. Chemerin is known to be susceptible to proteolysis by serine and cysteine proteases (Wittamer et al., 2003, 2005; Zabel et al., 2005a; Kulig et al., 2007, 2011). If chemerin serves as a local target for these proteases, p4 may be less likely to be degraded, which could preserve its bactericidal activity.

In addition to their antimicrobial properties, certain AMPs can trigger detrimental proinflammatory immune responses. LL-37, for example, engages nucleic acid-dependent proinflammatory signaling pathways that lead to autoinflammatory responses in the skin (Lande et al., 2007; Yamasaki et al., 2007; Ganguly et al., 2009; Zhang et al., 2019). Our data suggest that p4 does not exhibit apparent proinflammatory activity, as evidenced by reductions in skin-infiltrating leukocytes in *S. aureus*-infected and p4-treated AD skin (Figures 5D,E). Therefore, despite the lower anti-staphylococcal activity of p4 compared to that of LL-37 observed *in vitro* (Figures 1A,B), p4 may be attractive as a therapeutic agent against skin infection with MRSA, owing to its ability to inhibit *S. aureus* growth without triggering potentially pathogenic proinflammatory effects. However, this needs to be tested further.

MRSA such as USA300 is the leading cause of community-acquired skin infections (Carrel et al., 2015). Likewise, AD afflicts millions worldwide (Byrd et al., 2017; Kim et al., 2019). Skin colonization with *S. aureus*, particularly MRSA, hampers management of AD (Kim et al., 2019). *S. aureus* colonizes the skin of a majority of AD patients (60–100%). Approximately 10–30% of *S. aureus* cultivated from AD patient skin are methicillin resistant, particularly in association with previous patient hospitalization and usage of antibiotics to contain bacteria (Byrd et al., 2017; Kim et al., 2019). p4 markedly limited the growth of USA300 in experimental skin infection models, including experimental AD skin (Figure 4). Concomitant treatment of *S. aureus* with both p4 and LL-37 had an additive effect *in vitro* (Figure 1C), suggesting that these agents cooperate for optimal bacterial growth restriction. Of note, a decline in LL-37 and other AMP levels is a risk factor for chronic colonization of AD skin with *S. aureus* (Nakatsuji et al., 2017). Therefore, lower levels of bioactive cathelicidin could account for the diminished efficacy of p4 in eczematous skin. However, in our AD model, we did not observe a decrease in *camp* mRNA levels. Although we cannot exclude the possibility that differences in functional instead of merely expression levels of CRAMP contributed to the p4 effect, p4 may rely on other *in vivo* factors to better restrict *S. aureus* in uncompromised skin compared to AD-like skin. However, even partial inhibition of *S. aureus* USA300 growth by p4 resulted in reduced skin inflammation, as evidenced by lower levels of infiltrating leukocytes.

In conclusion, our data demonstrate that synthetic p4 can strengthen natural protection of skin barrier against MRSA, providing a potential therapeutic benefit to *S. aureus*-mediated skin inflammatory disorders.

## Data Availability Statement

The raw data supporting the conclusions of this article will be made available from the corresponding author upon reasonable request.

## Ethics Statement

The animal study was reviewed and approved by the Second Local Ethical Committee on Animal Testing at the Institute of Pharmacology Polish Academy of Sciences in Krakow.

## Author Contributions

AZ, UG, DK-C, PM, BZ, and JC conceived and designed the experiments. AZ, UG, DK-C, and PM performed the experiments. JC and BZ wrote the manuscript. All authors contributed to the article and approved the submitted version.

## Conflict of Interest

The authors declare that the research was conducted in the absence of any commercial or financial relationships that could be construed as a potential conflict of interest.

## Publisher’s Note

All claims expressed in this article are solely those of the authors and do not necessarily represent those of their affiliated organizations, or those of the publisher, the editors and the reviewers. Any product that may be evaluated in this article, or claim that may be made by its manufacturer, is not guaranteed or endorsed by the publisher.
